# Optimization-Based Motion Generation for Buzzwire Tasks With the REEM-C Humanoid Robot

**DOI:** 10.3389/frobt.2022.898890

**Published:** 2022-06-03

**Authors:** Peter Q. Lee, Vidyasagar Rajendran, Katja Mombaur

**Affiliations:** Human-Centred Robotics and Machine Intelligence Lab, Department of Systems Design Engineering, University of Waterloo, Waterloo, ON, Canada

**Keywords:** humanoid, buzzwire, optimal control, trajectory optimization, hardware, robot

## Abstract

Buzzwire tasks are often used as benchmarks and as training environments for fine motor skills and high precision path following. These tasks require moving a wire loop along an arbitrarily shaped wire obstacle in a collision-free manner. While there have been some demonstrations of buzzwire tasks with robotic manipulators using reinforcement learning and admittance control, there does not seem to be any examples with humanoid robots. In this work, we consider the scenario where we control one arm of the REEM-C humanoid robot, with other joints fixed, as groundwork for eventual full-body control. In pursuit of this, we contribute by designing an optimal control problem that generates trajectories to solve the buzzwire in a time optimized manner. This is composed of task-space constraints to prevent collisions with the buzzwire obstacle, the physical limits of the robot, and an objective function to trade-off reducing time and increasing margins from collision. The formulation can be applied to a very general set of wire shapes and the objective and task constraints can be adapted to other hardware configurations. We evaluate this formulation using the arm of a REEM-C humanoid robot and provide an analysis of how the generated trajectories perform both in simulation and on hardware.

## 1 Introduction

The buzzwire task is an agility based task where one holds a wire loop that encloses a long metallic obstacle, with the goal of moving the loop from one end of the obstacle to the other end without the two colliding. The name buzzwire refers to the electric version of the setup where each contact between loop and obstacle closes a circuit and results in a buzzing sound. Electric and non-electric variations of the buzzwire task are often incorporated into games for children (Köcher and Holzmüller, 2019), but they are also used as tests and training environments of motor skill and coordination (Budini et al., 2014; Bloch et al., 2015; Mann et al., 2018). The task can also be taken from a competitive standpoint, where one attempts to complete the task as fast as possible, as was featured in the 2016 Cybathalon (Degeler, 2016) as part of the prosthetic hand competition.

The buzzwire task has seen some implementations in the realm of robotics as well. Dorussen (2021) applied reinforcement learning on a fixed robotic arm to learn how to navigate the obstacle. Another work by Meyes et al. (2018) used the task as a basis for training a deep reinforcement learning policy. The task was featured in the work by Žlajpah (2017) to demonstrate a method of orientation control based on the task space of a two-arm robot. Žlajpah and Petrič (2019) again investigated the task from an admittance control perspective where a human guides a fixed arm to complete the task. More generally, tasks like the buzzwire task are important to the field of robotics because the precise motions that are required are applicable to many different high precision scenarios such as manufacturing tasks (Olsder and Suri, 1980; Pepyne and Cassandras, 2000; Xie et al., 2020). Optimal control provides a powerful framework for solving tasks that require high precision in a generalizable manner. There are many examples in the literature of optimal control problems that are designed to generate or implement a path constrained trajectory (Bobrow et al., 1985; Constantinescu and Croft, 2000; Verschueren et al., 2016; Shen et al., 2018). Among the different approaches, direct multiple shooting (Bock and Plitt, 1984) remains a reliable way of solving these problems (Diehl et al., 2006).

To our knowledge, there are no examples in the literature of humanoid robots accomplishing the buzzwire task. In our scenario, we consider the control of the arm of the REEM-C robot as groundwork for future full body buzzwire tasks. Therefore, the contribution of this work is:

• The formulation of an optimal control problem (OCP) that generates trajectories offline by way of parameterizing the obstacle shapes and formulating mathematical constraints to characterize the collision criteria between the obstacle and the end-effector loop *via* coplanar constraints. The OCP has an objective function that controls the trade-off between completing the task quickly and making the end-effector’s path more robust to collision. Such trade-offs are of particular importance to strike in a humanoid context where there are greater disturbances, due to stabilizers and positioning, compared to a stationary arm.
• We validate the proposed method on three different obstacles. By summarizing the results among different variable initializations, we show that our constraint formulation generates trajectories that run without collisions and demonstrate how the terms of the objective function can control the trade-off between total time and robustness to collisions.
• We evaluate and discuss the discrepancy between playing the trajectories on hardware versus simulation. We also identify challenges that come from performing the task on the hardware and discuss strategies to alleviate the issues that will be pursued in future work.

## 2 Methods

**FIGURE 1:**
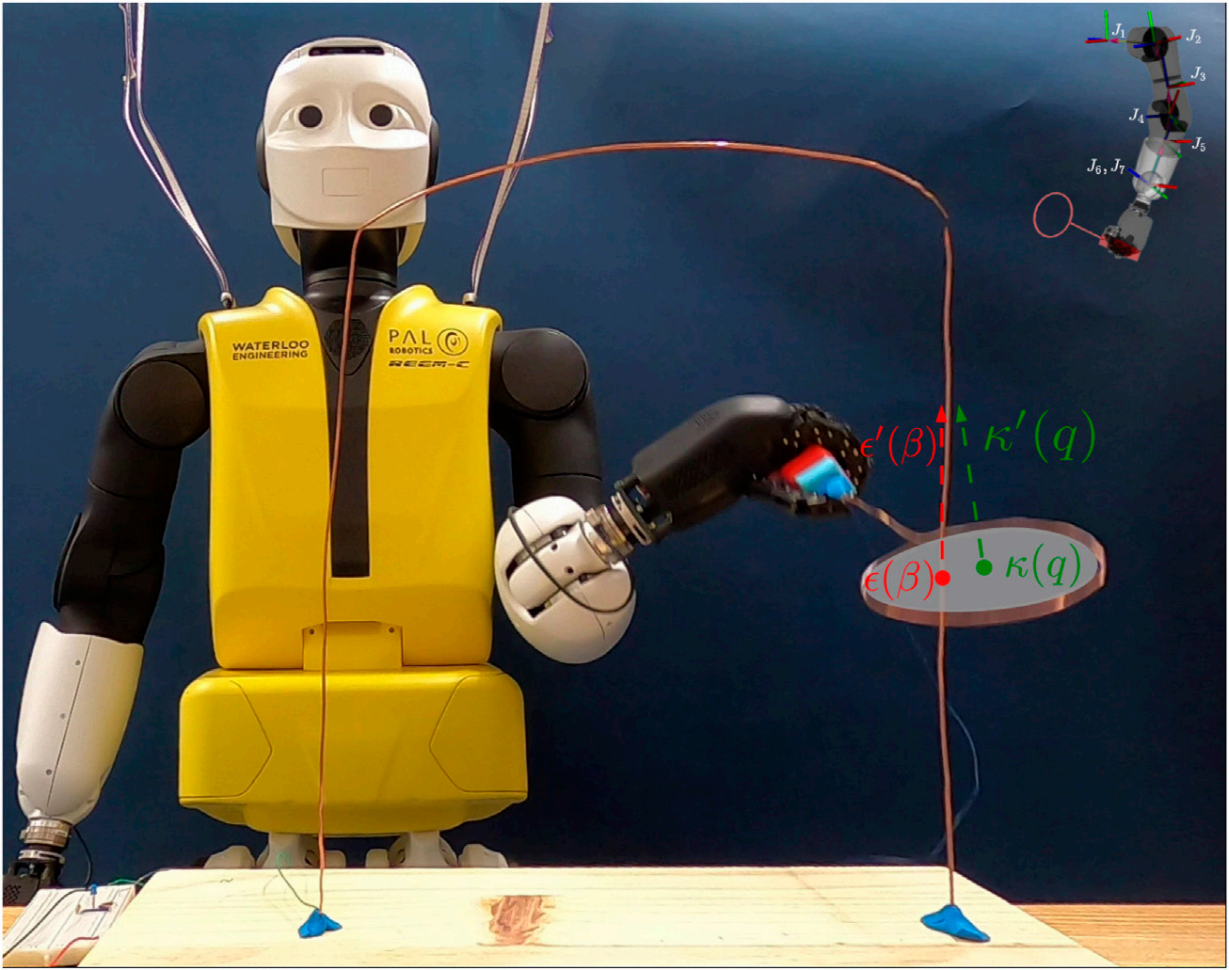
REEM-C grasping the loop end-effector and moving it along the buzzwire obstacle with an illustration of the end-effector intersecting the obstacle. The loop plane circle is in gray, with the centre, *κ*(*q*), and normal, *κ*′(*q*), in green. The reference point, at position *ϵ*(*β*), and the tangent, *ϵ*′(*β*) (in red) move along the wire. In the top right corner, the left arm of the REEM-C is pictured with joint axes labelled *J*_1_ through *J*_7_.

In this work, we use the humanoid robot REEM-C “Seven” that we have in our lab at the University of Waterloo. The humanoid is manufactured by PAL Robotics, Barcelona, Spain. Using its Hey-5 hand (Catalano et al., 2014) it firmly grasps the end-effector containing a copper loop, as shown in Figure 1.

### 2.1 Modeling the Robot

We model the motions of the robot arm holding the loop end effector as a rigid-body 7-DoF articulated manipulator whose equations of motion can be written as the system of ordinary differential equations (Siciliano and Khatib, 2016):

$$M\left(q\right)\ddot{q}+C\left(q,\dot{q}\right)\dot{q}+g\left(q\right)=\tau ,$$ (1)

where the vector $q\in R^{7}$ is the joint positions, $\dot{q}\in R^{7}$ is the joint velocities, $\ddot{q}\in R^{7}$ is the joint accelerations, $M\left(q\right)\in R^{7\times 7}$ is a positive definite mass matrix, $C\left(q,\dot{q}\right)\in R^{7\times 7}$ is a matrix of centrifugal and Coriolis effects, $g\left(q\right)\in R^{7}$ is a vector of gravitational terms and $\tau \in R^{7}$ is a vector of joint torques applied by the manipulator’s actuators. Coulomb friction and viscous friction are neglected in these equations of motion. Note that the low-level controllers for the REEM-C are kinematically driven, so joint accelerations are the primary input for the problem while the limits on joint torques are verified in the constraints to ensure that the motors can perform the motion.

We define the function $\kappa \left(q\right)\in R^{3}$ as the centre position of the loop end-effector following from the robot’s kinematics (including the loop end-effector tool) and $\kappa^{\prime }\left(q\right)\in R^{3},\|\kappa^{\prime }\left(q\right){\|}_{2}=1$ as the normal vector that is perpendicular to the 2D plane the loop is aligned with.

### 2.2 Wire Obstacle Parameterization

We assume that the obstacle is represented with cubic splines that interpolate among a set of discrete control points along the shape of the obstacle. The splines are parameterized according to normalized arc length, *β*, so that *β* = 0 is the start of the obstacle and *β* = 1 is the end. This has an advantage over directly parameterizing the obstacle with respect to time because it allows the solver to adjust the rate of traversal of the obstacle so it can adapt to different segments. So, for example, it can slow down at tight corners and speed up on easier straight segments. Therefore, the function $\epsilon \left(\beta \right)\in R^{3}$ describes the Cartesian coordinates of the points along the obstacle, while the function $\epsilon^{\prime }\left(\beta \right)\in R^{3},\|\epsilon^{\prime }\left(\beta \right){\|}_{2}=1$ is the normalized tangent vector of the obstacle at *β*. We further parameterize *β* with respect to time, along with taking its first $\left(\dot{\beta }\right)$ and second $\left(\ddot{\beta }\right)$ derivatives as additional control variables.

### 2.3 Optimal Control Formulation

Letting *t*_*f*_ be the total time taken to execute the trajectory, our optimal control problem is summarized by the following non-linear program (NLP):

$$\underset{f,\ddot{q},\ddot{\beta }}{t}\left\{t_{f}+\int_{0}^{t_{f}}\left[\alpha \|\kappa \left(q\left(t\right)\right)-\epsilon \left(\beta \left(t\right)\right){\|}_{2}-\nu \kappa^{\prime }{\left(q\left(t\right)\right)}^{⊤}\epsilon^{\prime }\left(\beta \left(t\right)\right)\right]dt\right\}$$ (2)

such that

$$\ddot{q}=\frac{\dot{q}}{dt},\dot{q}=\frac{q}{dt},\ddot{\beta }=\frac{\dot{\beta }}{dt},\dot{\beta }=\frac{\beta }{dt}$$ (3)

$$q^{\min}<q\left(t\right)<q^{\max}$$ (4)

$${\dot{q}}^{\min}<\dot{q}\left(t\right)<{\dot{q}}^{\max}$$ (5)

$${\ddot{q}}^{\min}<\ddot{q}\left(t\right)<{\ddot{q}}^{\max}$$ (6)

$${\overset{\ldots }{q}}^{\min}<\frac{\ddot{q}\left(t+\Delta t\right)-\ddot{q}\left(t\right)}{\Delta t}<{\overset{\ldots }{q}}^{\max}$$ (7)

$$\tau^{\min}<\tau \left(q\left(t\right),\dot{q}\left(t\right),\ddot{q}\left(t\right)\right)<\tau^{\max}$$ (8)

$$\dot{q}\left(0\right)=0,\dot{q}\left(t_{f}\right)=0,\beta \left(0\right)=0,\beta \left(t_{f}\right)=1,\dot{\beta }\left(t\right)\geq 0.0$$ (9)

$$-\delta <\kappa^{\prime }{\left(q\right)}^{⊤}\left(\kappa \left(q\right)-\epsilon \left(\beta \right)\right)<\delta$$ (10)

$$\|\kappa \left(q\left(t\right)\right)-\epsilon \left(\beta \left(t\right)\right){\|}_{2}<\rho$$ (11)

$$\kappa^{\prime }{\left(q\left(t\right)\right)}^{⊤}\epsilon^{\prime }\left(\beta \left(t\right)\right)>\mu .$$ (12)

In the spirit of the agility task, the objective, Eq. 2, is designed to minimize the time taken to complete the task, with auxiliary terms, which will be explained below, that allow tuning the trajectory for robustness. The system dynamics are described by Eq. 3. As Eq. 3 takes the form of ordinary differential equations, they are actually implemented *via* their closed form quadratic formulas between the time step Δ*t* (e.g., $q_{i+1}=q_{i}+{\dot{q}}_{i}\Delta t+\frac{1}{2}{\ddot{q}}_{i}\Delta t^{2}$ ). The constraints of the robot are included through Eqs 4–6, 8, which are the joint position, velocity, acceleration, and torque limits of each of the seven joints. To accommodate the non-instantaneous input response (i.e., avoid bang-bang solutions), Eq. 7 adds jerk constraints. Finally, Eq. 9 ensures that the joint motions start and end at rest and the path parameter starts at 0 and ends at 1 (restricting it from moving backwards in the sequence).

A unique aspect of the buzzwire task is that the wire obstacle must be contained within the loop end-effector and ideally it should be centred. To relate the intersection of the end-effector centre, *κ*(*q*), and the target obstacle point, *ϵ*(*β*), Eq. 10 is added to enforce that these two points are coplanar on the end-effector normal *κ*′(*q*), which would normally be represented through the equation *κ*′(*q*)^*⊤*^(*κ*(*q*) − *ϵ*(*β*)) = 0. Eq. 10 is given a slack of *δ* = 10^−4^ for numerical purposes during optimization. Next, Eq. 11 is implemented to ensure that the distance between *κ*(*q*) and *ϵ*(*β*) does not exceed a certain threshold. Because *κ*(*q*) and *ϵ*(*β*) can be assumed to be co-planar, Eq. 11 represents the distance of the two points within the circle. We note that *ρ* cannot exceed the radius of the circle, else a collision will occur. Figure 1 provides an illustration of the points relative to the end-effector and the obstacle. As an auxiliary optimization term, $\int_{0}^{t_{f}}\alpha \|\kappa \left(q\left(t\right)\right)-\epsilon \left(\beta \left(t\right)\right){\|}_{2}\;dt$ is added to the objective function Eq. 2 as an option to reduce the euclidean distance to increase trajectory robustness.

Another important aspect in this problem is constraining the orientation of the end-effector relative to the obstacle. As the normal vector of the end-effector, *κ*′(*q*), becomes orthogonal to the obstacle tangent, *ϵ*′(*β*), the edges of the circular end-effector get closer to the obstacle, reducing the tolerance to perturbations that may lead to collisions as compared to when the vectors are parallel. At the same time, some amount of angular tolerance is required between the two vectors to account for maneuvering around corners of the obstacle. Taking this into consideration, Eq. 12 ensures that *κ*′(*q*) and *ϵ*′(*β*) remain aligned so that *κ*′(*q*)^*⊤*^ *ϵ*′(*β*) does not drop below *μ* = 0.55 (i.e., the angle between these two vectors cannot exceed 56°). Therefore, the objective function Eq. 2 contains the term $\int_{0}^{t_{f}}-\nu \kappa^{\prime }{\left(q\left(t\right)\right)}^{⊤}\epsilon^{\prime }\left(\beta \left(t\right)\right)\;dt$ to bias the vectors to remain parallel. While the auxiliary terms described above are used to reduce the task-space errors, it is also possible to add similar terms to reduce joint acceleration, jerk, or torque as well. This would have the effect of making the arm motions more smooth and easier to run on hardware.

The joint limits *q* ^min^ and *q* ^max^ were taken from the URDF (Unified Robot Description Format) provided by PAL Robotics. The controllers on REEM-C bounded the joint velocity limits to ${\dot{q}}^{\min}=-1.5$ rad/s, ${\dot{q}}^{\max}=1.5$ rad/s. The acceleration and jerk limits were determined experimentally. We found that the hardware was much less generous than the limits specified in simulation, with large errors occurring if the limits were left too high. Likely, joint tracking errors and non-ideal step responses of the actuators required the more conservative limits. Ultimately, we found that ${\ddot{q}}^{\min}=-1.0$ rad/s^2^, ${\ddot{q}}^{\max}=1.0$ rad/s^2^, ${\overset{\ldots }{q}}^{\min}=-2.0$ rad/s^3^, and ${\overset{\ldots }{q}}^{\max}=2.0$ rad/s^3^ resulted in trajectories that were suitable to run on the hardware. The torque limits *τ* ^min^ and *τ* ^max^ were based on the effort limits provided by the URDF. For the problem specific constraints, we chose *ρ* = 0.01 m, since remaining centred along the wireframe is important to avoid collisions.

The optimal control problem (Eqs 2–12) is discretized using the direct multiple shooting method (Bock and Plitt, 1984), where each of the discretized shooting nodes were partitioned evenly throughout the duration. We used a total of 100 shooting nodes in our problem to ensure that the path constraints were continuously satisfied. The number of shooting points has an impact on both the accuracy of the solution and the computational complexity. More shooting points will ensure that the constraints are satisfied at more points of the trajectory, but will also add additional variables to the NLP and hence make the solver run for longer. We used the CasADi framework (Andersson et al., 2019) to setup the NLP, along with the Interior Point Optimizer (Wächter and Biegler, 2006) using the MUltifrontal Massively Parallel sparse direct Solver (Amestoy et al., 2019, 2001). The Rigid Body Dynamics Library (Felis, 2017) was used to compute the forward kinematics (*κ*) and the inverse dynamics (*τ*), using RBDL-Casadi bindings (Michaud et al., 2021) to ensure gradients could be calculated through the kinematics functions for the NLP solver.

## 3 Results and Discussion

With the optimal control problem described, we will now analyze the solutions on three different obstacles. We first define the physical setup for the problem, then discuss the implications of variable initialization, analyze different configurations in simulation, and finally compare the gap between simulation and hardware.

### 3.1 Physical Setup

**FIGURE 2:**
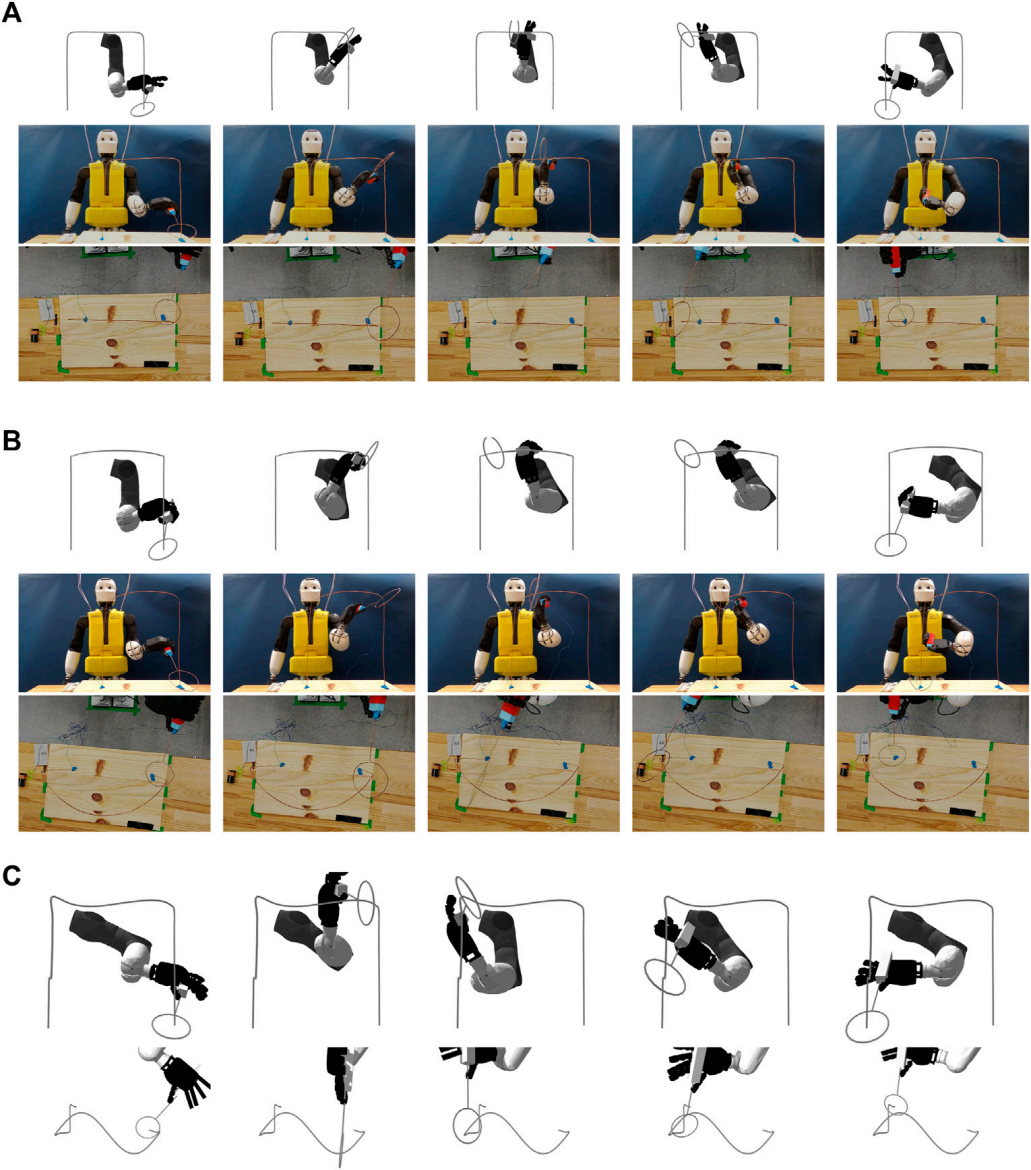
**(A)** Frames from the optimized trajectory on Obstacle-A executed by the REEM-C arm in the Gazebo simulator (top row) and the same trajectory executed on the full REEM-C humanoid hardware from a front view (middle row) and top-down view (bottom row). **(B)** Frames from the optimized trajectory on Obstacle-B executed by the REEM-C arm in the Gazebo simulator (top row) and the same trajectory executed on the full REEM-C humanoid hardware from a front view (middle row) and top-down view (bottom row). **(C)** Frames from the optimized trajectory on Obstacle-C executed by the REEM-C arm in the Gazebo simulator (top row) and the same trajectory executed on the full REEM-C humanoid in the Gazebo simulator from a top view showing the sine shape on the top portion of the wireframe (bottom row).

Physically, the end-effector was made with 1.6 mm diameter copper wire that protrudes 13 cm from the handle and is tapered with a 5 cm radius circular loop. We designed three obstacles for the end-effector to traverse. Obstacle-A, as shown in Figure 2A, has a right angled arch-like structure, that is parallel to the Y-plane, with each side length being 30 cm long. Obstacle-B, as shown in Figure 2B, has a similar height, but has a radial arc out of the Y-plane with a radius of 17.5 cm. Obstacle-C, as shown in Figure 2C, is the most complex, consisting of a sinusoidal pattern and additional bends out of the Y-plane. The cubic splines for these obstacles and a video overview of the trajectories can be found in the Supplementary Material. We constructed obstacles A and B by bending the 1.6 mm diameter copper wire according to printed projections of the structure. However, we did not construct obstacle-C because the wire was not rigid enough to support its out of plane bends; instead, obstacle-C was only evaluated in simulation and therefore serves as a theoretical benchmark. While the obstacles may seem simple from the perspective of a coordinated human, one must take into account the available workspace in the hardware. The REEM-C arm has a workspace in front of the robot that has a volume of about 40 cm × 40 cm × 40 cm. Consequently, the obstacles that are present approach the limits of what the arm can do without enabling full body motion.

### 3.2 Variable Initialization

One aspect that has an impact on the solution is the choice of initial variables along the shooting nodes for the NLP solver. We found that setting the joint positions to arbitrary values (e.g., *q* = 0) resulted in the solver becoming stuck at infeasible points (i.e., a point where the problem constraints are violated and the solver cannot find a way to approach a point where the constraints are satisfied). An inverse kinematics algorithm is needed to initialize *q* to a configuration that is closer to meeting the task space constraints to help convergence. But there are challenges in transplanting IK solutions as initialization points for the NLP. First, any joint configuration that satisfies the task space constraints along the obstacle is not unique. This is because the task is constrained to 5 degrees of freedom (DoF) by way of the 3-DoF euclidean constraint (Eq. 11), plus the 2-DoF orientation constraint (Eq. 12), since the dot product still leaves rotation about *ϵ*′(*β*) undetermined. As there are 7-DoF in arm, the IK solutions cannot be unique. Second, is the challenge of reachability in the choice of IK solution. If we take an IK solution that satisfies the constraints at one point of the obstacle, the ability to reach subsequent points on the obstacle is not guaranteed to exist when we consider the joint limits and the singularity conditions of the arm. As a result of these notions, the solver may need to be restarted with different initializations before it finds a solution.

We leverage the TRAC-IK inverse kinematics solver (Beeson and Ames, 2015) to initialize *q*. Our strategy takes the IK solution for *β* = 0 to initialize *q* at all of the shooting nodes. We generate 50 different IK configurations by randomly varying the unconstrained rotational DoF about the axis of *ϵ*′(0), as described above. We observed that about 68 of 150 initializations converged while the remaining became stuck at infeasible points. This is not a large issue from the offline planning perspective this paper takes because one can simply restart the procedure with a new IK configuration. We also briefly examined using the RRT-Connect Kuffner and LaValle (2000) path planning algorithm to initialize the intermediate nodes. While the initialized states were able to converge successfully, the extra runtime needed for RRT-Connect to find a feasible sequence of *q* made it uncompetitive compared to our original approach. Having a method that can quickly initialize the joints to a feasible state prior to optimization will be useful for online trajectory generation and is therefore a topic to explore in future work.

### 3.3 Simulation Summary

In our analysis, we compare the solutions between three different versions of objective functions on each of the three obstacles. The three configurations of the objective function (Eq. 2) were one with (*α* = 0, *ν* = 0), that corresponds to the time-optimal objective, another with (*α* = 30, *ν* = 1) that has a moderate mix of reducing time and task space errors, and one with (*α* = 150, *ν* = 5) that has a heavier emphasis on reducing task space errors. The goal of this analysis is to determine whether the proposed objective serves its purpose of allowing a trade-off between minimizing time and reducing the chances of collision with the obstacle. Table 1 shows the results in simulation summarized over the 50 different initializations. The trajectories were generated on a computer with an AMD Ryzen 3700X CPU, with 32 GB of RAM on the Debian Linux operating system. The median of the computational runtime to generate the trajectories was 15 s for obstacle-A, 25 s for obstacle-B, and 42 s for obstacle-C.

**TABLE 1:** Summary of optimized trajectories over 50 different initializations. The median time (*t*_*f*_) and 95% translation threshold (γ*) ± their interquartile range are reported among the instances that converged. The *p*-values come from testing the change of *t*_*f*_ and γ* between (*α* = 0, *ν* = 0) to (*α* = 30, *ν* = 1) and (*α* = 30, *ν* = 1) to (*α* = 150, *ν* = 5), using Mann-Whitney U tests, assuming a threshold of significance of 0.05. The *p*-values were adjusted with Bonferonni corrections for the multiple tests done on γ* and *t*_*f*_, respectively.

Obstacle	*α*	*ν*	γ* (cm)	*p*-value	*t*_*f*_ (s)	*p*-value
A	0	0	0.036 ± 0.001	—	2.958 ± 0.079	—
A	30	1	0.040 ± 0.002	4 × 10^−9^	3.045 ± 0.169	4 × 10^−2^
A	150	5	0.045 ± 0.001	7 × 10^−8^	3.551 ± 0.013	7 × 10^−8^
B	0	0	0.034 ± 0.001	—	3.920 ± 0.021	—
B	30	1	0.038 ± 0.001	3 × 10^−8^	3.979 ± 0.047	1 × 10^−5^
B	150	5	0.039 ± 0.003	1 × 10^−2^	4.586 ± 0.048	7 × 10^−9^
C	0	0	0.027 ± 0.002	—	5.964 ± 0.063	—
C	30	1	0.031 ± 0.002	3 × 10^−4^	6.293 ± 0.303	1 × 10^−5^
C	150	5	0.034 ± 0.001	1 × 10^−6^	6.966 ± 0.487	9, ×, 10^−5^

Of the problems that converged, we note that the median *t*_*f*_ increases as the obstacle gets more complex. Between the three objective function configurations we observe that (*α* = 30, *ν* = 1) have longer execution times than (*α* = 0, *ν* = 0) and (*α* = 150, *ν* = 5) are longer than (*α* = 30, *ν* = 1), with statistical significance evaluated with Mann-Whitney U tests, with the threshold of significance set to *p* = 0.05. While these effects are consistent, the magnitude differs between the configurations. The (*α* = 30, *ν* = 1) configuration has a relatively small impact on time, while (*α* = 150, *ν* = 5) is more substantial in magnitude. The difference in time also varies among the obstacles. In the simpler obstacles A and B, the change in median time is less than a 10th of a second for (*α* = 30, *ν* = 1) and up to half a second for (*α* = 150, *ν* = 5). But for the more complex obstacle-C, we can see that the change in median time grows to about three-tenths of a second for (*α* = 30, *ν* = 1) and one second for (*α* = 150, *ν* = 5). Ultimately, the impact of this time trade-off will depend on how important it is to the task versus the chances for collision.

In terms of measuring robustness of the trajectories, we want to determine how much we can perturb the obstacle in the task space before a collision would occur. In this sense, we play back each of the solutions while randomly translating the obstacle by *γ*, a vector between 0 and 5 cm, and record whether a collision occurs. With 1,000 trials of translating the obstacle by different *γ*, we take γ* to be the maximum threshold of translation where 95% or more of the playbacks run without collision. In Table 1 we can see that γ* steadily decreases as the obstacle gets more complex. By observation, the number of corners and tight turns, particularly in obstacle-C, can substantially reduce the error tolerance for the trajectory. As with time, γ* changes significantly as the *ν* and *α* change in terms of Mann-Whitney U tests. With (*α* = 30, *ν* = 1), there is a proportionally large increase in collision margin, with only a small penalty in time. In contrast, we start to see diminishing returns with (*α* = 150, *ν* = 5), where the time penalty becomes more significant compared to the increase in collision margin. This shows the impact of choosing the weights in the objective function appropriately to balance the trajectory time with respect to robustness to collision. In this scenario, we consider (*α* = 30, *ν* = 1) to be a suitable compromise.

### 3.4 Simulation-Hardware Comparison

**FIGURE 3:**
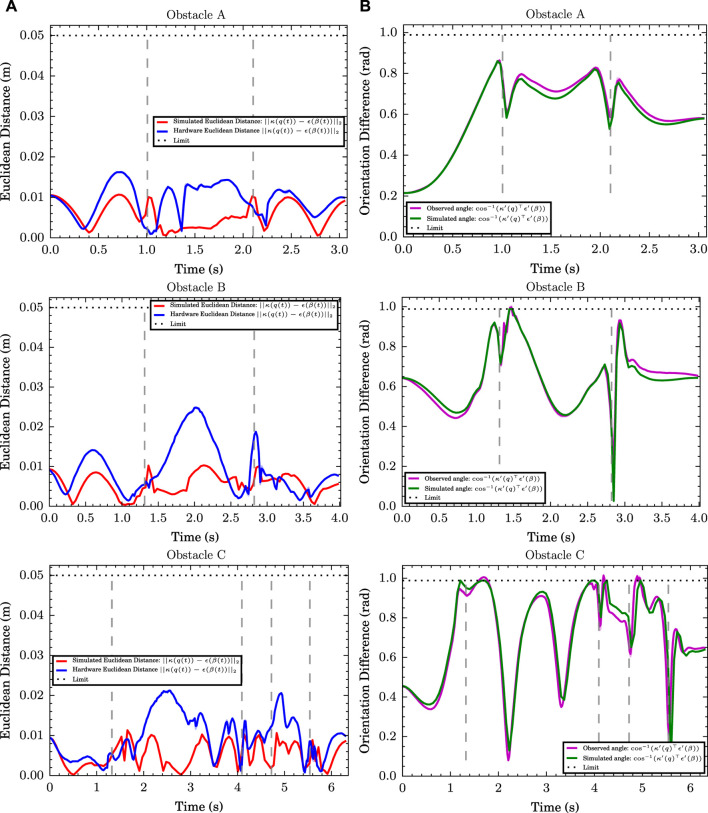
**(A)** Euclidean distance between the end-effector centre and the target obstacle position throughout the simulated (red) and hardware trials (blue) for obstacles A, B and C. **(B)** Difference in orientation between the obstacle tangent and the end-effector normal for obstacles A, B and C (simulations in green and experiments in magenta). The dashed grey lines indicate the time when the end-effector is at the sharp bend of the obstacle.

We now experiment with running the trajectories on the physical robot and compare how they differ from simulation due to the limitations imposed by the hardware. We selected example trajectories that were generated for all three obstacles using the *α* = 30, *ν* = 1 configuration and played them on the REEM-C robot holding the handle outside of the wire obstacles, averaging the results over 10 runs. Figure 3A compares the euclidean error of *κ*(*q*) and *ϵ*(*β*) between simulation and hardware, while Figure 3B shows the angular offset between *κ*′(*q*) and *ϵ*′(*β*). While the orientation corresponds rather closely, the hardware euclidean error deviates from simulation, particularly for obstacles B and C, as the joints struggle to keep up. One approach that could alleviate this is by adding joint acceleration, jerk, or torque penalty terms into the objective function, as suggested in Section 2.3, to make it easier for the motors to keep up, particularly when hardware limits are inaccurate. Future work will add these terms into the objective function and weigh the trade-off between reducing acceleration/jerk/torque, task space errors, and time minimization.

Despite this, the recorded hardware joint trajectory was still verified to be able to run without collisions if the obstacles were nominally positioned and constructed. A better analysis of hardware joint limits will be conducted in the future to more accurately set the constraints for the optimal control problem.

Finally, we ran the trajectories on the full physical setup with the end-effector enclosed in obstacles A and B, as shown in Figure 2. The obstacle formed an open-loop circuit so that if the end-effector made contact with the obstacle, the circuit would close and illuminate an LED. We were able to run the trajectories in both cases without making contact with the wire for both obstacles, but there were a number of challenges in getting these to work. As described in Section 3.3, the obstacle and robot had to be placed very precisely, with only a few centimetres of tolerance allowed before collisions would occur. Even with careful measurements between the obstacle and the robot’s base, variations in the joints of the legs and torso still have a significant impact on the placement and orientation of the shoulder. As mentioned above, inaccurate joint velocity limits meant that the wrist joints were not entirely able to track the trajectories. The loop on the end-effector needed to be oriented precisely to prevent collisions. Also, because the copper wire was non-rigid, the loop would oscillate during motion which could be a source of collisions. Finally, the construction errors from handcrafting obstacles is another difference from simulation.

In terms of extensions to other hardware configurations, our formulation can generalize to any configuration that can grasp an end-effector with a circular loop and have sufficient workspace to follow the obstacle through the 5-DoF task space constraints. Configurations with additional degrees of freedom will add to the problem’s computational complexity *via* adding more problem variables. Apropos of full body motion, the current formulation makes the assumption that the robot remains stable during motion; therefore extensions to full body motion will need to add additional constraints to ensure that the robot does not topple. The use of centroidal dynamics with full-body kinematics as in Dai et al. (2014), could be an attractive way to utilize full-body motion in a computationally feasible manner.

## 4 Conclusion and Perspectives

In this work, we have proposed an optimal control formulation for generating trajectories to accomplish the buzzwire task under a time-minimization strategy. The set of constraints proposed in the formulation model the limitations of the hardware and the criteria for collisions of the end-effector on the obstacle. By adding auxiliary terms to the objective function to reduce position and orientation error, we allow a trade-off in terms of speed and robustness from collisions due to disturbances in the hardware or the setup. We demonstrate that this formulation is effective in both simulation and hardware using the arm of the REEM-C humanoid robot.

A goal of this work was to lay the foundation for completing buzzwire tasks using the full body of humanoids. Therefore, future work will involve extending the number of controllable degrees of freedom to include the torso and legs. This would include 1) allowing for motions of the upper body and legs while standing, and 2) also allowing the robot to take steps and accomplish traversing obstacles of arbitrary length. More sturdy materials and precision will be needed in crafting the obstacle and end-effector to reduce the discrepancy between simulation and reality. The final extension would be performing the buzzwire from arbitrary, non-preplanned positions. This would need to have a very accurate system for estimating the obstacle’s shape (e.g., vision with depth sensing), as even a few centimetres of error will result in the trajectory failing.

## Data Availability

The original contributions presented in the study are included in the article/Supplementary Material, further inquiries can be directed to the corresponding author.

## Supplementary Material

Supplementary Video S1 A brief summary of the manuscript along with simulation and real hardware trial videos.

Supplementary Data Sheet S1 An HDF5 file containing the parameters for the splines used.
